# Intestinal Helminth Infections in Pregnant Women Attending Antenatal Clinic at Kitale District Hospital, Kenya

**DOI:** 10.1155/2014/823923

**Published:** 2014-05-27

**Authors:** A. W. Wekesa, C. S. Mulambalah, C. I. Muleke, R. Odhiambo

**Affiliations:** ^1^Department of Biological Sciences, Faculty of Science, Egerton University, P.O. Box 536, Njoro 20107, Kenya; ^2^Department of Medical Microbiology and Parasitology, School of Medicine, College of Health Sciences, Moi University, P.O. Box 4606, Eldoret 30100, Kenya; ^3^Department of Veterinary Clinical Services, Faculty of Veterinary Medicine & Surgery, Egerton University, P.O. Box 536, Njoro 20107, Kenya; ^4^Department of Gender Studies, Egerton University, P.O. Box 536, Njoro 20107, Kenya

## Abstract

Intestinal helminth infections during pregnancy are associated with adverse outcomes including low birth weight and prenatal mortality. The infections are a major public health problem in developing countries. A hospital based survey was undertaken for six months to determine the infection prevalence, intensity, and risk factors. The study involved expectant women attending antenatal clinic. Stool samples were screened microscopically for helminth ova using Kato Katz technique. Information on risk factors was collected using semistructured questionnaire and analyzed using SPSS. Epidemiological data was analysed using descriptive statistics and multivariate analysis. The overall prevalence of infection was 21 (13.8%). Ascariasis was the most prevalent 10 (6.5%), hookworm infection was 6 (3.9%), and trichuriasis was 2 (1.3%). Pregnant women aged below 29 years (OR = 3.63, CI = 0.87–11.75) and those with primary level of education (OR = 3.21, CI = 0.88–11.75) were at a higher risk of infection compared to those aged ≥ 29 years with secondary level of education. Hand washing was significantly associated with reduced likelihood of infection (OR = 0.18, 95% CI = 0.06–0.57). It was concluded that intestinal helminth infections were prevalent among pregnant women. We recommended that all expectant women visiting antenatal clinics be screened for intestinal helminth infections and positive cases be advised to seek treatment.

## 1. Introduction

Intestinal geohelminthiases are among the most common andwidespread of human infections/diseases in the developing world. They contribute to poor nutritional status, anaemia, and impaired growth [1]. Thousands of rural and impoverished villagers throughout the tropics and subtropics are often chronically infected with several different species of parasitic worms [2, 3]. About 2 billion harbour these infections worldwide, of which 300 million suffer associated severe morbidity and even death. The World Health Organization (WHO) estimates indicate that geohelminthiases account for more than 40% of the disease burden due to all tropical diseases.

Globally there are 800–1000 million cases reported of* Ascaris lumbricoides*, 700–900 million cases of* Necator americanus* and* Ancylostoma duodenale*, and 500 million cases of* Trichuris trichiura*. Although acute symptoms of infection are uncommon, numerous studies suggest consistent association between intestinal ascariasis and diminished food intake and weight loss [4]. The amount of work undertaken by a woman per day definitely declines when infected by geohelminths. In many agricultural communities, women often acquire helminth infections in the process of growing family's food.

Hookworm infection, whose prevalence and intensity vary by geographic region, is an important aetiological agent of anaemia in women of reproduction age [5]. Given that the geographical distribution of geohelminthiases widely overlaps in sub-Saharan Africa, the concurrence of hookworm infection during pregnancy may contribute significantly to the degree of anaemia in mothers and the newborn. The prevalence and intensity of infection are especially high in developing countries, among populations with poor environmental sanitation [6]. Other practices such as hand washing, disposal of refuse, personal hygiene, and wearing of shoes, when not done properly, may contribute to the infection or picking of infective forms of the worms from the environment [7].

A study by van Eijk and others [6] showed that in rural Western Kenya, intestinal geohelminthiases are common. Pregnant women are particularly vulnerable to infection, but the effect on pregnancy is not clear and may depend both on how heavy the worm infection is and on the type of worm species involved. In earlier studies, intestinal geohelminthiases were detected in three-quarters of 390 pregnant women in Western Kenya who provided a stool sample, and a prevalence of 76.2% was reported. In these women, the infections were associated with a modest decrease in haemoglobin levels and were indicators of poor nutritional status. Other studies in Western Kenya have reported a high prevalence of hookworm infection in comparison with* A. lumbricoides* and* T. trichiura* infections [8, 9].

Geohelminthiases are transmitted through the practice of soil eating common amongst pregnant women in many communities in developing countries [10]. The high rates of infection among pregnant women are mostly indicative of faecal pollution of soil and domestic water supply around homes due to poor sanitation and improper sewage disposal [10]. Pregnant women are also at high risk of infection because of their close relationship with children [6, 11]. Recently, pregnancy has been associated with an increase in prevalence of* A*.* lumbricoides* and* T. trichiura* infections compared to nonpregnant women [12].

Previous surveys reported the prevalence of geohelminthiases among pregnant women in Kenya [6, 9]. However, the studies did not adequately address prevalence and intensity of geohelminthiases in expectant women. The challenge has been exclusion of this group from deworming programs without considering the ratio. The study was undertaken to determine the prevalence and intensity of geohelminthiases in relation to the predisposing factors among pregnant women. The purpose of the study was to enhance epidemiological data on geohelminthiases and advance appropriate recommendations that could be undertaken to reduce the burden of infection and associated adverse pregnancy outcomes.

## 2. Materials and Methods

### 2.1. Study Area

The study was conducted at Kitale District Hospital located in Kitale town, with an estimated population of 20,000 people as per 2009 census. Kitale is located between latitude 1°01′58′′ north and longitude 35°00′02′′ east. It consists of slum settlements such as Kipsongo, Folk land, and Shimo la Tewa. The residents can be categorised as town residents and rural set-ups such as Bikeke, Cherangany, Kiminini, and Moi's Bridge.

### 2.2. Study Design and Subject Recruitment

The study was a hospital based survey in which consecutive sampling was used to recruit participants based on required criteria. The target population recruited in the study were pregnant women aged between 18 and 45 years, seeking antenatal services at Kitale District Hospital and who resided in the study area. The subjects who admitted to have received antihelmintics in 3 months prior to the study were excluded from the study. All subjects had to give written consent to participate in the study.

### 2.3. Sample Size Determination

The required sample size for this study was calculated based on 95% confidence level and 5% marginal error; sample size (*n*) was determined using the formula as described by O. M. Mugenda and G. A. Mugenda [13]. The minimum sample size (*n*) was calculated thus:

(1) $$\begin{array}{c} n=\frac{Z^{2}P\left(1-P\right)}{D^{2}}, \end{array}$$

where *D* is margin of error (0.05), *n* is the minimum sample size, *P* is the estimated prevalence (11.2%), *Z* is the standard normal deviate that corresponds to 95% confidence interval (1.96), and *n* = (1.96)^2^ × 0.112(1 − 0.112)/0.05^2^ = 152.828 rounded up to 153.

### 2.4. Specimen Collection and Processing

A sample of fresh stool specimen was collected from all the 153 participants. Subjects were provided with a labeled leakproof stool container (polypots), toilet paper, and applicator stick. Approximately 5 gm of stool specimens was collected into polypots, using applicator sticks. The stool specimens were examined microscopically within 24 hours of collection using the Kato-Katz technique. The procedure was preferred because it has been widely used to evaluate prevalence and intensity of intestinal infections and provides an accurate measure of the number of eggs present per gram of stool [14]. For each stool sample two Kato slides were made and the average of the total number of eggs was recorded. The magnifications of ×10 and ×40 were used, respectively, to visualize and identify intestinal geohelminth ova.

### 2.5. Kato-Katz Technique and Evaluation of Infection Prevalence and Intensity

Each stool specimen was prepared using a sieve and put on a calibrated template to weigh 47.1 mg of stool; this was used to calculate eggs per gram of faeces by multiplying eggs counted in the preparation by factor 50. The preparation on the glass slide was covered with glycerin/malachite green impregnated cellophane paper. The preparation was turned upside down on a flat surface and pressed gently to spread the stool sample before examination. The slides were examined within one hour to avoid over clearing of hookworm eggs. All eggs in each preparation were counted to determine the number of eggs per gram of faeces.

The egg counts were classified as light, moderate, and heavy infection as per WHO recommendations [15] as follows:* Ascaris*: light infection (1–4999 eggs/gram), moderate infection (5000–49,999 eggs/gram), and heavy infection (>50,000 eggs/gram); hookworm: light infection (1–999 eggs/gram), moderate infection (1000–9,999 eggs/gram), and heavy infection (>10,000);* Trichuris trichiura*: light infection (1–999), moderate infection (1000–9999), and heavy infection (10,000 eggs/gram).

## 3. Results

### 3.1. Prevalence and Intensity of Intestinal Helminth Infections

Overall prevalence was 21 (13.7%).* Ascaris lumbricoides* was the most prevalent 10 (47.6%), followed by hookworm infection by* Necator americanus* 6 (28.6%),* Trichuris trichiura* 2 (9.5%), and* Enterobius vermicularis* 1 (4.8%), and multiple infection of* A. lumbricoides* and* T. trichiura* accounted for 2 (9.5%). Pregnant women not infected were 132 (86%).

The intensity of intestinal geohelminthiases among (21) infected pregnant women was categorized as heavy 3 (14.3%), moderate 9 (42.9%), and low 9 (42.9%), respectively.

### 3.2. Socioeconomic Risk Factors Associated with Intestinal Helminth Infections

By use of a bivariate model age, waste disposal, hand washing, education level of the pregnant women, and type of housing were significantly associated with geohelminth infection. Younger pregnant women aged below 29 years had a higher probability of becoming infected compared to those aged over 30 years (85.7% versus 55.3%), respectively (*P* = 0.08). Women who did not use pit latrines for waste disposal were more likely to become infected compared to those who were using toilet as source of waste disposal (95.2% versus 4.8%) and the association was statistically significant (*P* = 0.047). A similar trend was also observed among those with primary education level where women with primary education level were at a higher risk of infection compared to those with secondary level of education (38.1% versus 9.8%), respectively. Participants who lived in permanent houses were less likely to become infected (14.3% versus 53.8%). Being employed or unemployed was not significantly associated with infection (*P* < 0.061).

At adjusted multivariate logistic regression model, younger pregnant women aged below 29 years (OR 3.63, CI 0.87–11.75) and those with primary education (OR 3.21, CI 0.88–11.75) were at a higher risk of infection compared to those who were older and had secondary level of education. Washing of hands was significantly associated with low likelihood of infection (OR = 0.18, 95% CI = 0.06–0.57).

## 4. Discussion and Conclusion

The prevalence and intensity of intestinal helminth infection among different populations are functions of many different factors, most importantly the environmental factors, parasitic factors, and host factors [16]. Intestinal geohelminthiases with* A. lumbricoides*,* Necator americanus*, and* T. trichiura* and* Enterobius vermicularis* were common among pregnant women attending antenatal services. These prevalence and intensity figures may be an underestimate of the true disease(s) burden because of the small sample size used.

All the parasites species encountered in the study area had been reported in other parts of Kenya [6, 9] and other parts of the world. In the study, an overall prevalence rate of 13.7% was reported and was higher than findings from previous related studies. For example, a study in Congo revealed 9% and in Nigeria 12.5%. The variation may be attributed to lack of efficient environmental sanitation observed in rural set-up in the study area.

The overall prevalence was lower compared to findings from other studies [11], in which prevalence of 19.6% among 827 pregnant women was reported in Nyanza Province of Kenya. In related studies a prevalence rate of 76.2% among 390 pregnant women was reported in rural Western Kenya [6], 45.1% in Brazil, and 69.7% in Indonesia. Fusein and others [17] reported prevalence of 23% among 300 pregnant women in Kassena-Nankana district in Ghana. Variation in the prevalence rates could be attributed to differences in sample size used and geographic area of study.

The predominance of* Ascaris lumbricoides* was comparable with previous studies [18, 19]. However, the 6.5% ascariasis prevalence was low compared to that reported in related studies [20] but significantly higher than 0–7% reported at a rural community in Mexico [21].

The high prevalence of ascariasis may be attributed to poor personal hygiene and low economic status. Further, the eggs of the parasite are known to adhere to dust, fruits, and even vegetables and due to poor observance of personal hygiene, pregnant women inadvertently get infected by eating these contaminated food items. Pregnant women in many rural areas depend on pit latrines for waste disposal with no facilities for hand washing after defecation. Such scenario accompanied by seasonal flooding and possible latrine overflow of human waste into drinking water sources and gardens is likely to lead to the high prevalence of ascariasis reported. Hand washing, higher education levels, and availability of modern water and toilet facilities were significantly associated with low likelihood of infection. These findings suggest that schooling and availability of appropriate sanitary facilities play an important role in the prevention and control of parasitic diseases.

Human ascariasis was a more common infection because of ease of spread through faecal pollution of soil, and so the intensity of infection depends on the degree of soil pollution [22]. Man acquires infection by accidentally ingesting embryonated eggs in contaminated food, drink, or soil.* Ascaris* ova are also spread by coprophagous animals and can thus be transported to locations far from the defecation sites [23]. The well-protected eggs can withstand drying and can survive for very lengthy periods in soil. This explains why the infection is distributed throughout the world, compared to other human intestinal geohelminth infections. Furthermore,* Ascaris* eggs are coated with mucopolysaccharide substance that enables them to adhere firmly to different surfaces [24].

Hookworm infection with* N. americanus* was the second most common geohelminth infection with a low prevalence rate of 3.9% compared to that reported in related studies in Kenya at 11.2% Kisumu [9] and 74.9% Kilifi [10]. Elsewhere the reported prevalence of hookworm infection in expectant women varies widely. Brooker et al. [10] reported 56.6% in the 128 pregnant women in Tanzania, 44.5% in the 2507 pregnant women in Uganda, and 8.1% in the 1038 pregnant women attending antenatal clinic in Venezuela. The differences in sample size in various studies possibly contribute to variations in prevalence.

The prevalence of trichuriasis at 1.3% was lower compared to 4.6% among 827 pregnant women in Nyanza Province of Kenya [9] and 1.7% reported in Nigeria [10, 25, 26]. The prevalence was however higher than 0.9% reported among pregnant women in Ghana [27]. Trichuriasis is specifically prevalent in the warm humid tropics where faecal contamination of the soil and water sources is a major factor in the transmission of the infection in a community. Transmission occurs through poor sanitary habits of indiscriminate defecation. Infections usually occur through ingestion of infective ova from contaminated hands, food, or drinks. Flooding and coprophagous animals play some part in the transportation of the ova to locations other than the defecation site. The low prevalence of trichuriasis reported in the present and related studies supports the claim that it is less common in the tropics. The differences in the prevalence may be due to environmental factors and sample sizes used in various studies.

The prevalence of enterobiasisat 0.7% was comparable to 0.7–1.0% reported in pregnant women in Ghana [27]. However, the prevalence of enterobiasis was low compared to 3.5% recent findings reported in Nigeria [28].

The study established that intestinal geohelminthiases are an underestimated public health problem among pregnant women and that socioeconomic factors play an important role in the establishment and spread of the infections in communities.

Intestinal geohelminthiases were more prevalent in pregnant women of 29 years and below compared to their older counterparts. Those with basic primary education and living in mud/semipermanent houses had higher chance of infection compared to their counterparts with secondary level education and living in permanent houses. Eating of soil and lack of regular hand washing contributed to infection. These findings were in agreement with other research related findings in Africa [9, 16].

Pregnant women living in rural areas had higher probability of becoming infected due to poor environmental sanitation, low socioeconomic status, and lack of appropriate methods of refuse disposal.

It is concluded that intestinal geohelminthiases are prevalent among pregnant women. Ascariasis, hookworm infection by* Necator americanus*, trichuriasis, and enterobiasis were the most prevalent infections in the study participants.

It is recommended that antenatal clinics should incorporate routine stool examination to detect parasitic infections in pregnant women and refer positive cases for appropriate treatment.
